# Impinging and Overriding Spinous Processes in Horses: A Narrative Review

**DOI:** 10.3390/ani15182679

**Published:** 2025-09-13

**Authors:** Nicola Pilati, Maria Chiara Pressanto, Angela Palumbo Piccionello, Francesco De Angelis Corvi, Francesca Beccati

**Affiliations:** 1 School of Bioscience and Veterinary Medicine, University of Camerino, via Circonvallazione 93/95, 62024 Matelica, Italy; angela.palumbo@unicam.it; 2 Sports Horse Research Center, Department of Veterinary Medicine, University of Perugia, via San Costanzo 4, 06126 Perugia, Italy; chiarapressanto.dvm@gmail.com (M.C.P.); f.deangeliscorvi@gmail.com (F.D.A.C.);

**Keywords:** equine back pain, kissing spine, thoracolumbar spine, impingement, over-riding, spinous processes

## Abstract

**Simple Summary:**

Overriding dorsal spinous processes (ORSPs) is a skeletal abnormality in horses characterized by the narrowing (<4 mm) of the interspinous space and, touching, or overlapping of the spinous processes. Kissing spine is widely recognized as a common condition in sports horses associated with chronic back pain and poor performance. This review wants to critically analyze the literature regarding this condition because despite its clinical implications, the true incidence of pain arising from ORSPs in the equine population and its pathogenesis remains speculative and poorly understood; moreover, the demonstrated lack of correlation between diagnostic imaging findings and clinical signs complicates the clinical scenario making treatment selection challenging.

**Abstract:**

The term overriding spinous processes (ORSPs), commonly known as “kissing spine syndrome,” refers to a skeletal abnormality in horses that is characterized by the narrowing (<4 mm) of the interspinous space and touching or overlapping of the spinous processes (SPs). This condition primarily affects the mid- and caudal thoracic vertebrae; however, ORSP can also affect the lumbar SPs. In the veterinary community, kissing spine is widely recognized as a common condition in sport horses, as it is often associated with chronic back pain and contributes to poor performance. Despite its significant clinical implications, the true incidence of pain arising from ORSPs in the equine population remains speculative and the demonstrated lack of correlation between diagnostic imaging findings and clinical signs complicates the clinical scenario. Over the years, several treatment approaches, both conservative and surgical, have been suggested to manage ORSP in horses. Although the development of these therapeutic strategies reflects ongoing efforts to address the complex nature of ORSP, the pathogenesis of the condition remains poorly understood. The aim of this review is to examine the literature to evaluate the current approaches to ORSPs and to highlight gaps in knowledge and directions for future research to improve the understanding, diagnosis, and treatment of this condition.

## 1. Introduction

The term overriding dorsal spinous processes (ORSPs), commonly known as “kissing spine syndrome,” refers to a skeletal abnormality in horses characterized by the narrowing (<4 mm) of the interspinous space and touching or overlapping of the spinous processes (SPs—often reported in the literature as dorsal spinous processes [DSPs] of adjacent vertebrae) [1,2]. This condition primarily affects the mid- and caudal thoracic vertebrae, particularly from the thirteenth thoracic vertebra (Th13) to the eighteenth thoracic vertebra (Th18), with the fifteenth thoracic vertebra (Th15) being the most frequently involved [3]. However, ORSP can also affect the lumbar SPs [4]. In the veterinary community, kissing spine is widely recognized as a common condition in sports horses, as it is often associated with chronic back pain and contributes to poor performance [5,6,7,8]. A recent survey of equine veterinarians [9] identified ORSP as one of the most frequently encountered pathologies in horses presenting with primary back pain. Interestingly, breed predisposition appears to influence the prevalence of ORSPs; Warmbloods, Thoroughbreds, and Quarter Horses seem particularly susceptible to developing this condition [10,11].

Clinical signs of ORSP can range from subtle behavioral changes to more complex performance issues, such as difficulty maintaining a canter, a reluctance to bend or move in certain directions, or struggling to perform specific movements [9,12,13]. Despite its significant clinical implications, the true incidence of pain arising from ORSPs in the equine population and its pathogenesis remains speculative and poorly understood. Radiographic and postmortem studies indicate that the anatomical prevalence of ORSPs can be as high as 92% [1,7]; however, it is well recognized that not all horses with radiographic changes exhibit clinical signs [2,14,15]. This underscores the complexity of the condition and emphasizes the necessity of a thorough clinical evaluation that extends beyond diagnostic imaging.

The demonstrated lack of correlation between diagnostic imaging findings and clinical signs complicates the clinical scenario [6], making treatment selection challenging. The aim of this review is to examine the literature from the last 25 years to evaluate the current approaches to ORSPs and to highlight gaps in knowledge and directions for future research to improve the knowledge, diagnosis, and treatment of this condition. We hope that our work will contribute to a deeper understanding of ORSPs and serve as a foundation for further research aimed at fully elucidating the pathogenesis of this condition and its true impact on equine performances.

## 2. Methods

Pubmed and Google Scholar Databases were searched for relevant scientific articles published from January 2000 to May 2025. The following terms were used in the search: “equine” and “horse” in combination with “spinous process”, “kissing spine”, “overriding dorsal spinous process”, “thoracolumbar spine” and “interspinal ligament”. The primary focus was on peer-reviewed literature published in the English language. All study types were included, except for doctoral dissertations and book chapters. Titles and abstracts were screened, and irrelevant papers were excluded from the literature pool. A cross-check of the references from the included studies was performed to identify additional research. The main text of the studies was also checked to ensure the integrity of this review. One hundred and seventy-five articles were assessed for eligibility, and seventy-seven of those were included in the final narrative review. Articles about other lesions affecting the thoracolumbar tract of the spine were rejected, as they were outside the scope of this review.

## 3. Anatomy and Functional Anatomy

The SP is one of the vertebral processes associated with each vertebra. It projects dorsally from the vertebral arch and varies in shape, length, and orientation along different vertebral regions. Its function is to serve as a lever attachment site for muscles and ligaments [16]. The first ten thoracic vertebrae (Th1–Th10) have SPs that progressively elongate and obliquely oriented caudally. From the thirteenth to the fifteenth thoracic (Th13-Th15) vertebrae, the SPs gradually shorten, and their orientation becomes increasingly vertical, reaching a perpendicular position at the anticlinal vertebra (Th15) [17]. The SPs caudal to the anticlinal vertebra are then obliquely oriented cranially and continue to gradually shorten in the lumbar and sacral regions [18]. The SPs are covered by the supraspinous ligament, a fibroelastic band that extends as a continuation of the nuchal ligament. This ligament stabilizes the apex of the SPs and aids in resisting excessive spinal flexion. It affects the dorsocranial aspect of each SPs and its composition varies throughout the thoracolumbar region. It is slightly elastic in the cranial thoracic vertebrae but becomes progressively more fibrous as it extends caudally toward the tuber sacrale [19]. This ligament consists of a dorsal part, which continues the thoracolumbar fascia, and a ventral fibrous part, which connects to the craniodorsal aspect of the SPs and receives fibers from the interspinous ligaments [20]. The interspinous ligament is a well-developed ligamentous structure, which connects two adjacent SPs along almost their entire height. This broad fibrous band also receives fibers from the multifidus muscle and the SPs’ periosteum [20]. It is composed of four sagittal layers: two abaxial layers that originate from the caudoventral aspect of the cranial SP and influence the craniodorsal aspect of the adjacent caudal one, and axial layers that run in the opposite direction [20,21]. Dorsally, the four layers fuse with the supraspinous ligament and distally, the abaxial layers are inserted close to the articular processes and joint capsule, especially in the thoracic spine. A histological study has demonstrated that there is no distinction between fibers of the interspinous ligament and supraspinuos ligament in the area where they merge and there is evidence about the presence of very few contractile elements within these two structures [20]. The SPs and related ligaments are surrounded by the epaxial and hypaxial muscles which contribute to stabilizing this section of the axial skeleton. The epaxial muscles of the equine thoracolumbar spine include the erector spinae muscle group, which is composed of the longissimus dorsi, the iliocostalis, the spinalis dorsalis, and multifidus muscles. These muscles are covered by the gluteus medius muscle on the most caudal portion of the thoracolumbar tract and in the lumbar area [22]. The hypaxial muscles consist of the ileopsoas and the minor psoas muscles, which function as flexors of the coxofemoral joint and also play a role in flexion of the spine and the pelvis when the four legs are fixed on the ground.

The innervation of all the structures of the thoracolumbar spine is guaranteed by thoracic and lumbar spinal nerves; the dorsal ramus of each of them provides sensory and motor innervation of the epaxial muscles and the soft tissues of the proximal aspect of the related vertebra and the ventral ramus to hypaxial muscles and associated skin [23]. A spinal nerve merges at the level of the intervertebral foramen, beginning with a meningeal branch followed by the dorsal ramus and the ramus communicans before continuing as a ventral ramus. The dorsal divides into a lateral branch, which runs caudolaterally in a 45° direction from the sagittal plane and innervates the epaxial muscles, and a medial branch that runs caudo-dorsally adjacent to the dorsal aspect of the SP of the vertebra innervating the epaxial muscles, which are soft tissues associated with the proximal aspect of the vertebrae and the skin [23]. One author noted that the interspinal ligament has dense sensory innervation [20] that, in humans, has been demonstrated to arise from the medial branch [24]. It has been described in humans, dogs and cats that an intermediate branch is present in the thoracolumbar spine [25,26], but this branch has not been described in horses in the thoracic region; it has only been detected in the lumbar region [27].

The interaction between bones, ligaments, muscles, and their connection to the rib cage is responsible for the complex and limited mobility of the thoracolumbar spine. In each region of the vertebral column, mobility is influenced by variations in the morphology of both osteoarticular and soft tissue components [17,18]. One study demonstrated the intimate anatomical connection between the supraspinous and interspinal ligaments, the thoracolumbar fascia, and the fascicles of the multifidus muscle [20]. The authors described similar histological fiber arrangements among these structures, suggesting that they may share a common biomechanical function in counteracting distraction forces in this segment of the axial skeleton.

The kinematics of the thoracolumbar spine can be described through six fundamental movements: three rotations (lateroflexion, flexion–extension, axial rotation) and three translations. However, because translational movements involve only minimal displacements between vertebrae, they will not be considered in the description of vertebral motion. The biomechanical behavior of the thoracolumbar region varies in relation to the movements, as demonstrated by both in vitro [28,29] and in vivo studies [30,31]. Several studies have shown that the flexion–extension range—defined as rotation around the sagittal axis—gradually decreases from the Th10 to the second lumbar vertebra (L2) [30]. The segment between Th14 and Th18 has been identified as the most flexible portion of the thoracolumbar spine [28,30], whereas the smallest flexion–extension movement occurs at both ends of the thoracolumbar region, specifically at Th3 and between the third and fifth lumbar vertebra (L3-L5) [32]. These differences in mobility are closely linked to the anatomical variation of the SPs. In the more mobile Th14–Th18 segment, the SPs are shorter and thinner, while in the lumbar region, they become progressively taller and thicker [29,30]. These morphological adaptations are accompanied by narrowing of the interspinous ligament and shortening of the supraspinous ligament. Additionally, the cranial thoracic spine features very tall SPs and articulates with the rib cage via sternal ribs. This anatomical connection to the sternum significantly limits mobility in this region [28]. The anatomy of the thoracolumbar spine also affects lateroflexion and axial rotation, which are most pronounced between Th9 and Th14 and diminish progressively in cranial and caudal directions [28,29]. During flexion, the SP of one vertebra moves cranially relative to the one caudal to it, creating tension in the interspinous and supraspinous ligaments. Extension results in the opposite motion [28]. Lateroflexion involves lateral shifting of the SPs and is coupled with contralateral axial rotation, which generates shearing forces on the interspinous ligaments and their insertions on the margins of the SPs. This movement places stress on the dorsal aspect of the SPs and associated soft tissue attachments, potentially contributing to pain in cases of impinging SPs or ORSP [23]. Lastly, movements of the thoracolumbar spine are also influenced by the position of the neck. When the neck is lowered, the nuchal ligament exerts traction on the most cranial SPs of the thoracic area, causing them to become more vertical. This results in elevation and flexion of the thoracic spine just beneath the saddle area [28].

While the above studies provide insights into equine axial kinematics, there is a lack of in vivo data evaluating how different equestrian disciplines affect thoracolumbar movement. Further research is needed to assess how specific exercises and training routines influence equine back kinematics. A better understanding of discipline-specific biomechanics could certainly improve the interpretation of clinical and diagnostic imaging findings and guide the management of horses presenting back pathologies, including ORSPs. Despite the high radiographic prevalence of ORSPs in Thoroughbreds, clinical signs are often absent or controversial. Based on both the literature [1,14,33] and the authors’ clinical experience, it is plausible that the type of exercise performed in flat racing contributes to this phenomenon. Flat racing typically involves repetitive flexion–extension, mainly at the lumbosacral junction, with limited lateral or rotational loading. The thoracolumbar spine is further stabilized by strong epaxial musculature, and the rider’s weight is positioned forward, away from the saddle area. This biomechanical configuration may allow Thoroughbreds involved in flat racing to better tolerate ORSP lesions compared to horses in disciplines such as show jumping or dressage, where a greater range of complex spinal movements is required and it could explain the absence of clinical signs in some Thoroughbreds with clear radiographic evidence of ORSPs, provided there are no concurrent osseous pathologies such as vertebral body spondylosis or articular facet joint osteoarthritis.

## 4. Etiology

**Figure 1:**
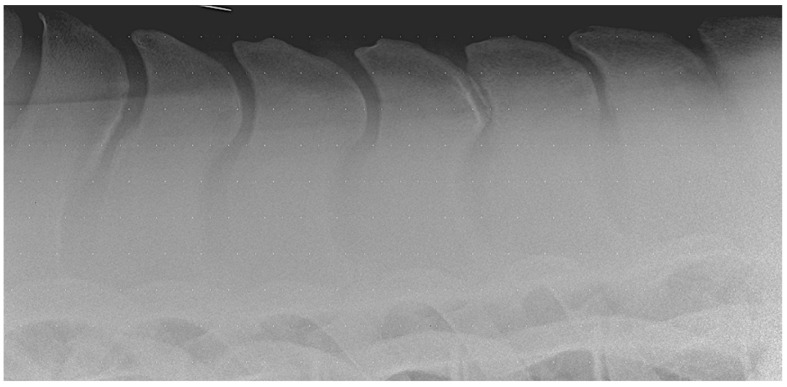
Lateral radiographic image of the mid-thoracic area obtained in a 15-year-old Warmblood gelding with kissing spine at Th15-Th16. Note the peak-pick summits of the spinous processes.

The etiology of ORSPs is complex, multifactorial, and not yet fully understood. Most of the existing literature supports the hypothesis that ORSPs are more likely acquired rather than congenital in origin. Indeed, a radiographic study on Warmblood foals found no evidence of narrowing of the interspinous spaces up to three months of age [32]. Several factors have been proposed in relation to the acquired development of ORSPs, including age, sex, vertebral column biomechanics, and breed predisposition [34]. In particular, the greater range of motion observed in the caudal thoracic region—especially in athletic horses—has been considered a contributing factor. Some authors suggest that the repetitive mechanical stress and overload associated with exercise may lead to the development of these lesions. Conformational traits, such as a short back or specific SP morphologies (e.g., “peak-pick summits”), particularly in certain breeds (e.g., Thoroughbreds), have also been proposed as potential risk factors [1,34,35] (Figure 1).

**Figure 2:**
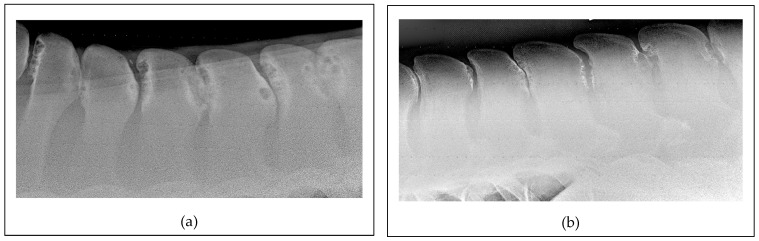
Lateral radiographic projections of the mid-thoracic area: (**a**) An image obtained for a 20-month-old Thoroughbred colt. Note the severe presence of narrowing, overriding, and kissing spine lesions between Th12 and Th17 with extensive osteolysis and sclerosis. The horse had never been ridden prior to the radiographic examination. (**b**) An image obtained for a 20-year-old Thoroughbred mare used for racing at the beginning of the life and then for pleasure riding and low-level show-jumping. In this case also, there is severe narrowing, overriding, and kissing spine lesions between Th15 and L3 with extensive osteolysis and sclerosis.

Additionally, improper saddle fit and rider-induced pressure may cause repetitive trauma and inflammation, further contributing to the development of acquired ORSPs [36]. More recently, a study identified a significant correlation between the severity of ORSP lesions and a specific single-nucleotide polymorphism, indicating a possible genetic component [10]. This finding opens up a promising direction for future research aimed at developing preventative strategies and reducing the incidence of ORSPs in the equine population. Further challenging the purely acquired condition theory, one author recently reported a comparable prevalence and severity of ORSPs in Thoroughbred yearlings and adult horses (Figure 2) [2]. Notably, these young horses had not undergone formal training and had only engaged in spontaneous field activity.

**Figure 3:**
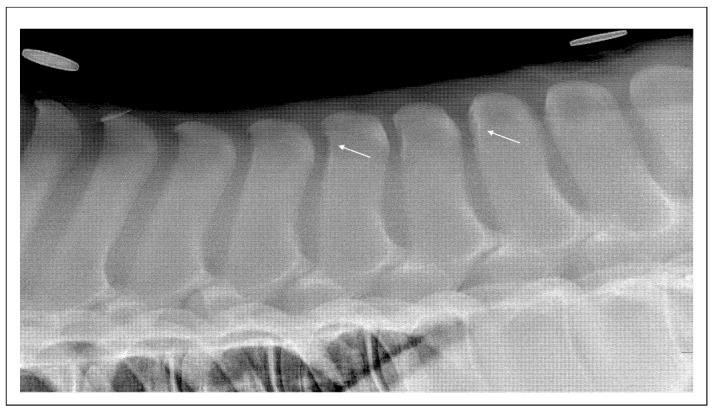
Lateral radiographic projection of the thoracolumbar junction in a 186-day-old Thoroughbred filly. There are linear small radiolucent areas surrounded by mild sclerosis (white arrows) of the cranial aspect of Th15 and T18.

These findings suggest that at least some ORSPs may originate earlier in life, independent of external workload (Figure 3).

Based on this and other observations, we propose a developmental pathogenesis for ORSPs, involving genetic predisposition, rapid growth, and nutritional imbalances—features shared with other developmental orthopedic diseases (DODs) observed in foals. These elements need better clarification from future research and should prompt further investigation to clarify the developmental nature of ORSPs. Long-term follow-up longitudinal studies are warranted to elucidate how radiographic lesions progress under different exercise regimens and in various breeds, and to explore the heritability of the condition, as well as the influence of environmental factors such as diet and management practices during early life.

## 5. Clinical Signs and Diagnostic Imaging Findings

### 5.1. Clinical Signs

ORSPs are considered one of the causes of primary back pain in horses [1,7,37].

**Figure 4:**
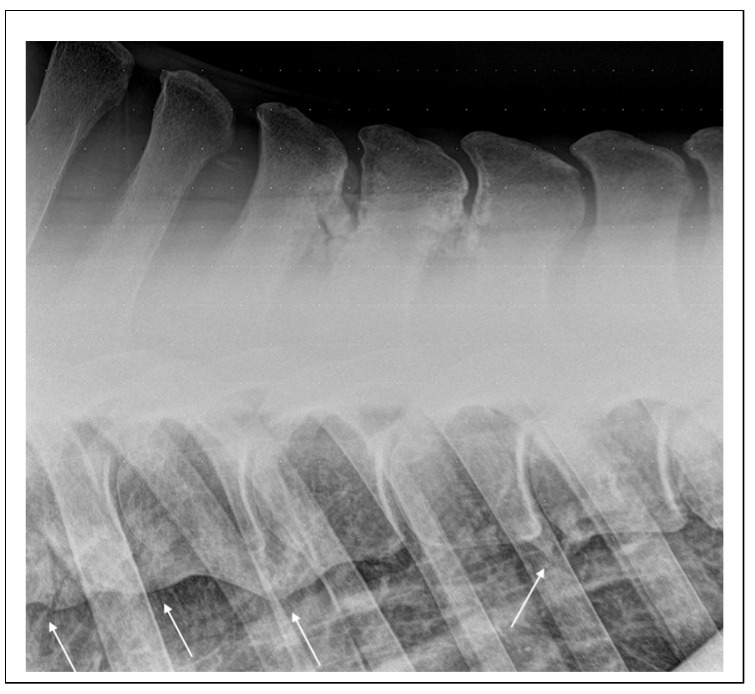
Lateral radiographic projection of the mid-thoracic area obtained in a 20-year-old Dutch pony used for dressage that was referred for abnormal behavior during ridden exercise and suspected back pain. There are extensive mineralizations of the interspinal space between Th11 and Th13 and advances spondylosis involving the vertebral bodies of Th10-T12 and Th13-Th14 (arrows).

In the literature, studies specifically addressing back pain primarily related to ORSPs in horses are notably scarce. Instead, research tends to focus on primary back pain as a broader category, encompassing various pathological alterations of the equine spine. This generalized approach often groups different conditions under a single umbrella, making it challenging to isolate the clinical and diagnostic features specific to ORSPs. Furthermore, existing studies predominantly involve adult horses, in which ORSPs are likely to co-occur with other pathologies, such as osteoarthritis of the articular process joints [38] or, less commonly, with spondylosis (Figure 4).

This overlap complicates the differentiation of clinical signs and the attribution of back pain to a single source. Given these limitations, this section adopts a broader perspective by reviewing back pain in horses with ORSPs.

Horses with suspected back pain possibly related to ORSPs of the thoracolumbar spine generally present with vague and nonspecific clinical signs [39,40]. These may include resistance to grooming or saddling, hypomobility of the spine during ridden exercise, poor hind limb engagement or jumping technique, reluctance to work, or, more generally, lack of performance [6,8,9,12,41,42]. Clinical examination is crucial in evaluating the presence of back pain and linking it with diagnostic findings of ORSPs. However, such evaluation has a high degree of subjectivity. A logical starting point should be ruling out any other potential causes of lameness or poor performance, thereby supporting the axial skeleton as the primary source of clinical concern [1,9,41].

Although several protocols for clinical assessment of the equine back have been described, no universally accepted state of the art currently exists. This lack of standardization contributes to variability in assessment and interpretation among clinicians [40,41,43,44]. Moreover, no objective measurement techniques are widely available in routine clinical settings.

Evaluation of the back’s conformation and the development of the epaxial muscles, along with palpation of the SPs, muscles, and soft tissues of the back, and performing passive mobilization of the thoracolumbar spine, are standard procedures among equine practitioners that are used to conduct a thorough evaluation of the equine back. These steps should always be carried out to ensure a comprehensive evaluation of the back. However, the specific methods used for palpation and mobilization usually vary enormously, often relying on the clinician’s experience and personal preferences. Two surveys conducted among clinicians in Europe and the USA stated that palpation and passive mobilization were considered the two most reliable procedures for assessing back pain [8,9].

Dynamic examination should also be performed, with the horse observed under different conditions—such as walking in a figure-eight, trotting in a straight line, lunging at trot on both reins and on both hard and soft ground, and cantering on soft ground.

This allows the clinician to identify reductions in flexion, extension, or lateral bending of the thoracolumbar spine under different conditions. Despite this, recent findings showed poor inter-observer agreement between clinicians (veterinarians and physiotherapists) and between clinician and objective kinematic data with regard to the evaluation of a horse walking and trotting in a straight line [45]. These results highlight the continued reliance on subjective clinical judgment and underscore the need for the development of objective methods to assess spinal mobility and dysfunction.

Efforts to standardize and objectify clinical assessments have been explored, though their use remains limited in routine equine practice. One such method is the use of an algometer—a device that quantifies mechanical nociceptive thresholds at specific points along the back. This technique has been applied in both human and equine studies with reasonable intra-individual repeatability [11,46,47]. However, significant inter-individual variability in response to nociceptive stimuli and the possible adaptation to pressure stimuli overtime [46] limit its usefulness for population-wide standardization.

Objective gait analysis systems (i.e., Lameness Locator) based on wireless inertial sensor systems have been described as useful tools for objectively assessing lameness in ambulatory practice. However, further research is required to validate the use of objective gait analysis systems for the evaluation of primary back pain [45].

Evaluation of the horse being ridden should always follow static and dynamic examination because this situation can highlight any abnormal behavior related to horse–rider interaction; of course, this has to be related to the affected horse’s possible reluctance to be ridden, making this evaluation possibly dangerous for the rider [1,48].

### 5.2. Diagnostic Imaging Findings

Imaging of the back—particularly radiography and scintigraphy—is a cornerstone in the diagnosis of ORSPs, alongside ultrasonographic examination, which complements radiography and is especially valuable for ultrasound-guided therapeutic and diagnostic procedures [11]. Accurate interpretation of imaging findings, in correlation with clinical examination, is essential to avoid over-interpretation that could lead to misdiagnosis and unnecessary treatments.

Radiographic images of the SPs of the equine back can be readily obtained using latero-lateral projections, even with modern ambulatory equipment, which nowadays enables the acquisition of high-quality images [41]; however, evaluating deeper structures of the equine thoracolumbar spine, such as articular processes or vertebral bodies, requires a high-output X-ray generator, which is typically available only in a hospital setting [13]. As a result, ambulatory clinicians may be limited to a partial radiographic assessment of the equine back, increasing the risk of misinterpretation and potentially leading to inaccurate diagnosis or suboptimal treatment.

Various grading systems to evaluate the SPs have been described and are commonly used by practitioners to assess radiographs of the equine back [2,6,34,49]. However, a definitive cut-off grade that clearly distinguishes between a sound horse and one with clinically manifested pathology has yet to be established. This is likely because such a cut-off may never exist, as it is closely tied to individual differences and their subjective tolerance to pain. As a result, achieving absolute certainty in this distinction remains inherently elusive. Moreover, many authors underlined that several abnormal radiographic findings of the SPs can also be detected in horses that are performing normally [2,14,50] and correlation between clinical signs and imaging findings is not always reliable [1,13,34]. Ultrasonography is a diagnostic technique useful for evaluating the proximal third of the SPs, as well as the interspinous and supraspinous ligaments, epaxial muscles, and thoracolumbar fascia [40,45]. Using either a linear or a convex probe, narrowing of the interspinous space, remodeling and bone bridging of the caudal and cranial margin of two adjacent SPs can be easily detected and graded in detail to compare them with the radiographic findings using a sagittal and paramedian approaches [11,51,52].

Nuclear scintigraphy is considered part of the multimodal approach to examining and imaging a horse’s back [1,41]. The presence of abnormal findings is not always associated with clinical manifestations, and has also been observed in clinically sound horses [14,34,46,50,53]. For this reason, several authors have emphasized the importance of interpreting clinical, radiographic and scintigraphic findings to correlate them with true back pain [1,35,49]. Despite ongoing research, there is currently no consensus on how to interpret imaging findings and in relation to the clinical signs. Consequently, clinicians must often rely on their own clinical experience, as misinterpretation of scintigraphic images may lead to diagnostic errors. Further studies involving larger populations and appropriate control groups are needed to clarify this critical aspect of ORSPs diagnosis; nowadays, this is still difficult because the lack of standardization of clinical examination in horses affected by back pain leads authors to interpret results differently, making the comparison of studies difficult and the creation of homogeneous and useful control groups non-consistent.

### 5.3. Diagnostic Analgesia

Diagnostic analgesia of the thoracolumbar spine can be considered as a diagnostic tool that can help with the evaluation of the clinical significance of the imaging findings as the real source of pain in horses [12,37,42,48].

The use of a 20G, 4 cm needle directed straight into the affected interspinal space, or immediately adjacent to it, to inject a volume of 4 to 10 mL or more of anesthetic solution was used to create analgesia of the affected area with or without radiographic control of the placement of the needle [6,37]. One author [54] compared different methods and volumes of solution for the injection of the ISP of the thoracolumbar tract; their research realized that the use of a 20G 4 cm needle and 5 mL of solution with a midline approach straight into the ISL both with ultrasonographic guidance or by palpation is the best method to avoid consistent diffusion of the solution on the surrounding structures. Regardless, some diffusion of the solution, especially on the multifidus area on the side opposite to the operator, was inadvertently achieved. The described position of the needle, considering the nerve anatomy of the area, could easily diffuse the analgesic solution close to the medial ramus of the dorsal branch, thus desensitizing the adjacent soft tissues structures making the block less specific, especially if several SPs have been blocked together. The results of one study demonstrated that horses with clinically significant ORSPs histologically presented malalignment of the fibers and modification of the ligamentous layers of the interspinal ligament associated with an increase in the nerve fibers within it compared to the ligament of normal SPs [21]. This histological background could justify the pain associated with kissing spines and the response of the horse to diagnostic analgesia of the area, but further studies have to be performed in asymptomatic horses with radiographic evidence of ORSPs to understand if similar changes are present in the interspinal ligament. This scenario has to be borne in mind when interpreting the clinical response to the analgesia of ORSPs. The use of diagnostic analgesia was successfully compared to the outcome after surgical treatment in one study [7], while the results from another study were controversial [6]. However, local injection of anesthetic solutions in the interspinal space of the thoracolumbar spine has been shown to alter spinal kinematics (proprioception) even in clinically sound horses [55]. Therefore, the interpretation of diagnostic analgesia results requires caution and clinical context. Interestingly, diagnostic analgesia was reported to be used infrequently by a large group of clinicians participating in a survey both in Europe [9] and US [8], highlighting the ongoing controversy surrounding its diagnostic value and variability in clinical application. The development of an ultrasonographic-guided technique to better guide the accuracy of the needle toward the lesions and consequently inject a very low volume of local anesthetic could be an option to avoid diffusion of the analgesic effect to close structures gaining specificity in the interpretation of radiographic findings.

## 6. Treatment and Rehabilitation Approaches

Conservative, medical, and surgical treatments have all been described for the management of impinging or ORSPs. However, the effectiveness of a stand-alone treatment option is often limited, and a multimodal approach is typically required [56]. In addition, studies about adjunctive therapies had a short-term outcome and the long-term function is unknown. When looking at surgical treatments, the long-term outcome is often incomplete and not specific for each sports discipline. The primary goal is to break the pain cycle, thereby allowing the horse to regain core strength and restore physiologic back function.

Medical management is often the first-line approach for treating back pain and includes local injection of corticosteroids, homeopathic preparations or distillates of *Sarracenia purpurea*, mesotherapy, or the systemic administration of bisphosphonates, all of which are aimed at reducing local inflammation and alleviating pain at the lesion site [8,9,55,57,58]. One study demonstrated that local medical treatment successfully resolved clinical signs of SP impingement, but symptoms reoccurred in the long term [7]. According to a survey conducted in Europe [9], systemic anti-inflammatory drugs were commonly used in the early 2000s for managing back pain, but their use declined significantly over the following decades due to limited long-term efficacy. When horses fail to respond to medical therapy, surgical treatment becomes the preferred approach for many [7,37].

Several surgical techniques have been described for approaching ORSP, including resection of the SPs (partial, complete or minimally invasive ostectomies) or the more recently described desmotomy of the interspinous ligament. The aims of these procedures are to eliminate/avoid/reduce the contact between the ORSPs. Some authors have described the resection of the summit of the affected SPs with an open technique in general anesthesia that considered creating a midline division of the supraspinous ligament to access the SP [59,60,61]. Another author used the same technique in a group of standing and sedated horses, eliminating the risks of general anesthesia and decreasing the amount of hemorrhage in the surgery field [62]. An endoscopic, minimally invasive approach under general anesthesia has been described [63] in a small group of horses, in which it was used to partially resect impinging or ORSPs with the result of a smaller incision than those created in the abovementioned studies.

Another surgical treatment of kissing spines is desmotomy of the interspinous ligament [7,64]; this technique consists of a small paramedian incision where a mayo scissor is inserted to resect the interspinous ligament, avoiding an open technique. The results described a long-term improvement in the racing results after desmotomy of the interspinous ligament on a large group of Thoroughbreds, mainly steeplechasers, compared to a control group of similar athletes [64]. In this study, the patient group was accurately selected after evaluation of the history and clinical and diagnostic imaging findings that confirmed the back pain was caused by ORSPs; diagnostic analgesia was not used to confirm the diagnosis and conservative treatment was used as a first step but proved unsuccessful in the long term [64]. Even if this is the only study that compares a patient population to a matched control group for long-term follow-up, the results are encouraging and would indicate the need for surgical treatment when a diagnosis of ORSPs is made and conservative treatment is not successful in the long term. This study included only Thoroughbreds used for steeplechase, and so similar studies, with matched controls, should be performed on horses racing in flat races or competing in other disciplines to better understand the outcome after surgical treatments. A summary of all the papers that described a surgical technique and its outcome are summarized in Table 1.

Interestingly, the results of a large survey [9] indicate that the ambulatory practitioners did not report surgical treatments as options for their caseload in Central southern Europe, and a survey on a population of US vets reported that 70% of enrolled clinicians would consider surgery only when medical treatment did not result in the expected improvement and also that most of the horses that underwent surgery for ORSPs did not improve in the short term after surgery and still required treatments during long-term follow-up [8]. All these data are interesting because views on the preferred approach likely differ based on the geographical position of the vets and the discipline they are used to working with: people from the UK and Ireland are more prone to implementing surgical approaches at an earlier stage, especially with patients used for flat racing and national hunt, while in the other parts of Europe, especially for sports horses, clinicians tend to use different medical treatment approaches.

It is well known that both medical and surgical treatments always go together with adjunction therapies that act on the pain threshold and help to rebuild the strength of the epaxial musculature [56]. Various therapies have been described in the literature for the treatment of back pain, including shockwave therapy, low-level laser therapy, acupuncture, kinesiotaping, and therapeutic ultrasound and their effect on the management of back pain (Table 2).

Most of the time, few details on the underlying pathology are given to the reader and some studies lack control groups to validate the efficacy of the treatment compared to a placebo group. As a result, the true therapeutic effect of these modalities on ORSPs cannot be reliably extrapolated from the published literature. Further research—including well-designed studies with appropriate placebo-controlled groups—is needed to clarify the effectiveness of these treatments in horses affected by ORSPs.

Once the pain cycle of ORSPs has been broken by one or often more of the methods described above, it is paramount to rebuild stability and core strength to successfully prevent back pain in the long term. Horses, like humans, can be caught by the mechanism in which pain caused by ORSP lesions can influence and inhibit the use and development of deep spinal stabilizing muscles, thus resulting in a worse instability of the intervertebral unit that viciously predisposes the animal to further pathology and pain, resulting in a greater level of muscle atrophy. In the authors’ opinion, stabilizing the thoracolumbar tract of the spinal column is necessary to create or regain postural stability for a long-lasting soundness after treatment of ORSPs. Different kinds of exercises have been described to achieve this goal by working on epaxial muscle strength (Table 3).

Many studies have reported the importance of the multifidus muscle in the stabilization of the thoracolumbar spine of humans and horses [65,66,67,68], and its hypertrophy has been reported to be associated with increased postural stability [68]. The picture we can create by reading the literature is incomplete: the standards for building a protocol of core exercises are not clear yet. Clinicians are not yet supported by studies with control groups, and most of the time, the population of patients used is not homogeneous between authors; moreover, back pain diagnoses are often vague and not supported by diagnostic imaging that could address the structures involved. Future studies involving larger numbers of patients, control groups, and a definitive diagnosis of the underlying condition are needed to give the clinician a better understanding of what the next steps should be.

**Table 2:** Summary of relevant papers regarding adjunctive therapies and their effect on the treated patients.

Study	Lesion	Type of Treatment	Group	Control Group(s)	Method Used to Assess Improvement	Conclusions	Reference
Xie et al., 2005	Generic thoracolumbar pain	Electro-acupuncture	12 horses with back pain; a total of 8 were treated with acupuncture	8 treated with phenylbutazone 4 with saline.	Thoracolumbar pain score assessed via clinical examination	Horses treated with acupuncture showed an improvement in their thoracolumbar pain score compared to other groups from the third treatment onward	[69]
Haussler et al., 2020	Generic back pain	Low-level laser therapy	61 quarter horse competing	No, 3 groups: laser, laser + chiropractic, chiropractic	Visual analog scale; clinical examination; algometer	Low-level laser therapy improved clinical signs of back pain, epaxial muscle hypertonicity, and truck stiffness	[70]
Trager et al., 2020	Back pain after clinical exam; X-ray of the back	Extracorporeal shockwaves	12 horses in sport activity	No	Clinical examination and back passive mobilization + algometer	Improvement in both algometer response and clinical examination	[71]
Mongkolrat et al., 2021	Back pain after clinical exam and positive acupoint scan	Laser stimulation of acupuncture point or therapeutic ultrasound	28 horses in training	No, horses were divided in two groups, one therapeutic U\S and one laser stimulation of acupuncture points	Algometer and acupoint sensitivity scans	Post-treatment back pain improvement, statistically better with laser stimulation of acupuncture points.	[72]
Garcia Piqueres et al., 2021	Patient without any recent history of back pain	Kinesiotaping	15 horses in moderate physical activity	No, two groups 1 with application of kinesiotape with tension and one without	Algometer + palpation over DSPs	Nociceptive threshold improved 60 min after application of tapes, and was better in the no-tension group	[73]
King et al., 2024	Epaxial muscle pain after clinical examination	Kinesiotaping	22 clinically affected horses involved in sport training	19 horses received both kinesiotaping and placebo in different times	Pressure algometer used to measure mechanical nociceptive threshold	Kinesiotaping group improved by up to 25% from baseline. The control group also showed an improvement of up to 6%	[74]

**Table 3:** Summary of relevant papers regarding core strength exercises and their effect on the treated patients.

Study	Lesion	Type of Exercise	Group	Control Group(s)	Method Used to Assess Improvement	Conclusions	Reference
Stubbs et al., 2011	Generic thoracolumbar pain	Dynamic mobilization exercises	8 Arabian sound horses not ridden during the rehab period	No	Cross sectional area (CSA) of the multifidus muscle assessed ultrasonographically	Hypertrophy of the M. multifidus in response to therapeutic exercises	[75]
Pfau et al., 2017	Sound horses	Use of elastic resistance bands	7 sport horses in full work	No	Eight inertial sensors on the back from the poll to the tail attachment	4 weeks of exercises improved back stability, especially of the thoracolumbar tract, on in-hand trot in a straight line and circle	[76]
Hasberghe et al., 2017	Lameness and back pain	Whole-body vibration (WBV)	9 sport horses with different degrees of lameness; 6 with back pathology and ORDSPs	No; CSA measurement started 30 days prior to WBV treatment to analyze the effect of just working on the CSA	CSA of the multifidus muscle in 4 different locations assessed ultrasonographically	Evident increase in CSA and left/right symmetry on multifidus muscle after 60 days	[77]
Fair et al., 2023	Sound horses	Walking on an inclined underwater treadmill	6 sport horses in training	No	Flexible curve ruler used in saddle-fitting kits; measurements taken at 4 points along the thoracolumbar tract.	Significant increase in epaxial muscle profile after the third week of exercises.	[78]
Ellis et al., 2024	Thoracolumbar pain	Whole-body vibration plate	10 sport horses diagnosed with thoracolumbar pain at palpation	No	Algometer; CSA of the multifidus; postural sway	After 30 days of daily use, multifidus CSA and postural stability increased	[79]

## 7. Conclusions and Direction for Future Research

Although ORSPs are considered to be a widespread condition and are often encountered both in sound and clinically affected horses, surveys of equine veterinarians have demonstrated that there is a lack of a tailored diagnostic workup and a standardized therapeutic approach to this condition. First of all, the literature indicates that the umbrella of ORSPs includes several degrees of the disease like close, impinging, overlapping, or fused SPs, and better clarification of the impact of every single degree of clinical manifestation would be helpful for interpreting clinical and diagnostic imaging findings. The paucity of pathognomonic clinical signs associated with an objective difficulty accessing the whole equine back with ambulatory equipment or advanced diagnostic techniques make the interpretation of clinical signs totally dependent on the experience of the clinician who lacks any objective methods for assessing the clinical soundness of the equine back. Despite decades of research, the therapeutic approach has not been standardized yet; the clinician will choose the treatment based on experience and/or rider preference without guidelines that can help him to choose the best multimodal treatment and rehabilitation exercises. A recent study [2] postulated that kissing spines could be considered as a developmental disease, and this could open up new scenarios for the interpretation of radiographic signs, especially in younger horses. A consensus must be reached regarding the interpretation of signs during clinical and diagnostic imaging examination, which would help to better standardize the approach to ORSPs and more general back pain even for research purposes; this would lead to a selection of a homogeneous population of patients in different studies that will allow us to compare results more objectively. Another topic that needs further research is adjunctive therapies and the rehabilitation approaches: nowadays, studies still do not provide clear guidelines and leave treatment decisions to the experience of the clinician and the rider; thus, objective methods extrapolated from studies with large and homogeneous populations compared to control groups are needed to create protocols that will guarantee the soundness and athletic health of sports and racing horses.

## Figures and Tables

**Table 1:** Summary of relevant papers describing surgical techniques to treat ORSPs and their outcome.

Study	Surgical Technique	Case Selection	Control Group	Results	Comments	Reference
Jeffcot and Hickman 1975	Resection of summits of ORSPs under GA with splitting of the SSL	Mixed population of horses affected by ORSPs pain based on clinical examination and radiographic grading; diagnostic analgesia used in few cases	No	11 out of 14 horses improved after surgery and returned to full activity	Poor objective definition of improvement after surgery and no control group	[60]
Desbrosse et al., 2007	ORSPs endoscopic resection under GA	Back pain or IDSP not responding to medical treatment diagnosed clinically radiographically and with local diagnostic analgesia	No	All horses returned to full work and initial complaints of the owner addressed	No objective definition of improvement after surgery; results based on owners’ opinion with no detailed information about pre- and post-surgery activity available	[63]
Coomer et al., 2012	Desmotomy of ISL by stab paramedian incision	Back pain based on clinical examination and radiographic findings, no local anesthesia, and no scintigraphy	1 group treated medically vs. 1 group treated surgically	Short-term follow-up similar for the 2 groups; long-term overall success of treatment was 46% in the medical group and 95% in the surgery group	Treatments not randomized; surgery group had more severe lesions. No clear definition of success. No data on the activity of the horses before and after treatment	[7]
Derham et al., 2019	Desmotomy of ISL by stab paramedian incision	Flat and national hunt racehorse with ORDSP pain diagnosed by clinical examination and radiographic score of ORDSPs; no local anesthesia	Control group with horses of the same age, trainer, and type of training	Comparison of performance pre- and post-treatment: surgery group showed improved performances after surgery compared the to control group	Efforts were made by the authors to minimize bias in the control group, but different training programs cannot be excluded. Only Thoroughbreds were included	[64]

## Data Availability

The raw data supporting the conclusions of this article will be made available by the authors on request.
